# Analysis of relationship between loading condition and cranial cracking pattern using a three-dimensional finite element model

**DOI:** 10.1186/s12891-022-05215-x

**Published:** 2022-03-31

**Authors:** Yoshimori Kiriyama, Yudai Sato, Yota Muramatsu, Teppei Mano, Katsumasa Tanaka, Kotaro Oshio

**Affiliations:** 1 Department of Mechanical Systems Engineering, Kogakuin University, Tokyo, Japan; 2 Course of Mechanical Engineering, Kogakuin University, Tokyo, Japan; 3 Kogakuin University, Tokyo, Japan; 4 Department of Neurosurgery, St. Marianna University, Kanagawa, Japan

**Keywords:** Skull fracture, Hairline cracks, Extended finite element method, Forming limit diagram

## Abstract

**Background:**

A hairline crack on the cranium can occur even under a small external load or impact and are thus often observed in patients who have experienced an accidental fall or collision. Typical finite element analysis is useful to analyze the stress concentration or the propagation of stress waves. However, a stress propagation model does not accurately reproduce the features of hairline cracks on the cranium. The objective in this study was to reproduce cranial hairline cracks.

**Methods:**

A three-dimensional finite element model of the cranial bone was developed from a patient CT images. The model consists of the frontal, parietal, occipital and temporal bones, and the bones are connected with the sutures. Additionally, the model comprised three layers; the external and internal tables and the diploe. The model was analyzed using the extended finite element method (X-FEM), and a forming limit diagram (FLD) was embedded in the model. In this study, the model was symmetrized bilaterally using the model developed from the left side of the skull. The FLD in this study was assumed to be a relationship between the maximum and minimum strains when a fracture occurs. A total of 13 typical loadings were applied to the model: loading points on the top, left, and back of the cranium were considered, and at each loading point, loads were applied with four or five different directions, namely perpendicular to the cranium and inclined in the anterior, posterior, superior, or inferior at an angle of 45^∘^.

**Results:**

Under all loading conditions, many small cracks formed radially at the loading points. Moreover, some large cracks formed under the certain loading conditions. The crack shapes on the top and left side could be associated with the specific loading directions, whereas cracks on the back did not show distinguishing characteristics depending on the loading directions. The present model was reproduced anatomically and morphologically, and the results were similar to those obtained in previous cadaver experiments.

**Conclusions:**

Through X-FEM analysis of the FE model embedded with an FLD, hairline cracks in the cranium were reproduced, and a few crack shapes were identified as potential markers for estimating the loading conditions.

## Background

A cranial fracture is a break in the cranial bones of the skull. Hairline cracks, which are one type of cranial fracture, are a common trauma of the skull[1] that can occur even under small external loads or impacts. Hairline cracks are often observed after a fall or collision or as a result of contact sports [2] and may also be caused by child abuse or domestic violence [3, 4]. These different causes of cranial fracture produce different mechanical loads, but it can be difficult to determine the cause of a fracture because the relationship between the hairline crack pattern and the mechanical loading conditions has not been fully elucidated. A general model of cranial fracture formation would aid in the identification of the causes and potential consequences of fractures in specific patients.

In an attempt to develop such a general model, Yoganandan et al. [5] have evaluated the relationship among the loading conditions, stress propagation and the patterns in the resulting cranial hairline crack using human cadavers. However, a single specimen cannot be used for repeated measurements, because a specimen with cracks is not intact. This complicates the elucidation of a general relationship between the crack structure and the mechanical conditions.

Another approach to this modeling effort is to develop finite element (FE) models of the cranium. To date, however, most FE studies have focused on the brain instead of cranial fractures because the brain is one of the most important vital organs. Therefore, stress propagation in the brain has been investigated using FE analysis of the head including the skull and the brain [6, 7]. FE analysis is also useful to analyze stress concentration or the propagation of stress waves along the skull. However, in previous investigations, the simulated stress propagation has not necessarily reproduced the observed cranial hairline crack. There are three large problems with this approach.

First, many FE models of the skull have not had sufficient anatomical precision. The skull is composed of eight cranial bones — the frontal, two parietal, two temporal, occipital, sphenoid and ethmoid bones — connected with the sutures. The sutures of the cranial bones represent structural discontinuities, and therefore affect the stress condition. However, in many FE analyses, the cranial bones have been modeled as one three-dimensional surface.

Second, in many past studies, the mechanical stress has been evaluated without considering the destruction of the bone. Even though high stress generally destroys the bone, it has not necessarily been considered that the stress can destroy the FE models. Comparison of the predicted stress with the yield stress is important to predict the occurrence of bone fracture.

Finally, related to the second point, FE models of the cranium do not typically include the necessary mechanisms to reproduce the crack location, because the crack does not necessarily follow the borders of the mesh elements in the FE model. To reproduce an arbitrary crack location in a FE model, the FE model must be equipped with a computational mechanism of cracking.

On this basis, the objective in this study is to reproduce the structure and location of cranial hairline cracks. For this, we used a three-dimensional FE model of the cranial bones. The model consists of the frontal, parietal, occipital, and temporal bones, and they are connected with sutures. The model was analyzed with the extended FE method (X-FEM)[8–10], and a forming limit diagram (FLD) was embedded in the model. An FLD shows the probability of fracture occurrence with respect to the maximum and minimum strains. In this study, the relationship between the loading conditions and hairline cracks in the cranial bones were evaluated using FE models.

## Methods

### Finite element cranial model

A female volunteer (age 30) with no history of trauma or clinical diagnoses was selected for the study. Informed consent was obtained in the form of opt-out online. The cranial CT image data were acquired using the imaging parameters given in Table 1. The three-dimensional surface of the cranial bones was obtained by segmentation using thresholding.

**Table 1:** CT aquisition data

Slice thickness [mm]	0.1
Resolution [pixel]	426×392
Slices	221

The borders of the sutures were segmented and were found to generally meander with variable widths. In this study, the widths of the sutures were measured in intervals of 5 mm along the suture direction and averaged across the suture. Based on the acquired widths, the sutures were separated from the other bony parts. In the cross-sectional area of the cranial bones, the diploe, which is the spongy bone, lies between the external and internal tables, which are the compact bones. As a result of the segmentation of the sutures, the thickness varies across the skull. To ignore the subject-specific structure, the bones in the model were defined as having a constant thickness; the external table was 1.7 mm, the diploe was 3.1 mm, and the internal table was 1.7 mm.

**Fig. 1:**
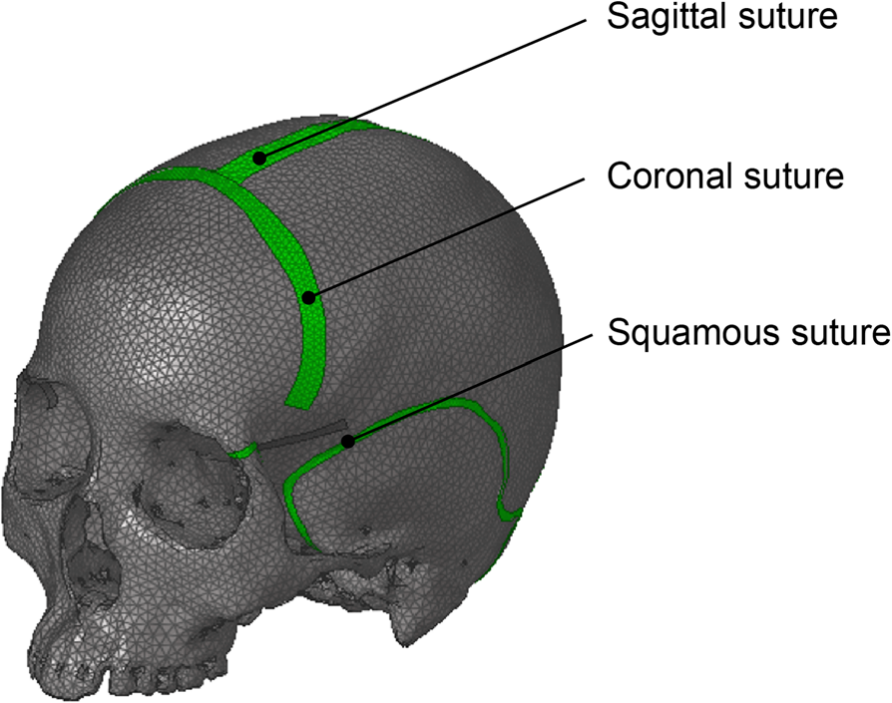
Three-dimensional finite element model of the skull

From these measurements, the cranial FE model was constructed using shell elements. In this study, the composite shell elements were used to represent three layers consisted of the external and internal tables and the diploe. The shell elements had not only the material properties as Young’s modulus but also the structural properties as the thickness. Figure 1 shows the FE model, and its material properties are given in Table 2. Anatomically, the sutures lie between two or more bony plates that engage in a complicated manner. The shapes of the sutures and the engagement of the bony plates are complex and vary from individual to individual. Hence, the sutures were modeled as bands whose width was determined based on the above measurements. Additionally, it was assumed that the suture engages with the compact material, and compact material properties were applied to the suture meshes.

**Table 2:** Material properties of the model

Material property	Ext./Int. tables	Diploe
Young’s modulus [×10^1^GPa]	1.5	0.1
Density [×10^3^kg/m^3^]	2.0	1.3
Poisson ratio [–]	0.22	0.24
Yield stress [MPa]	5.3	60.0
Thickness [mm]	1.7	3.1

In this study, a symmetric model was analyzed to reduce the effects of bilateral differences. Hence, the right side of the model was constructed by reflecting the left side across the median sagittal plane, and these two sides were connected. Here, the discontinuity of the nodes was modified, and then the surface was smoothed to maintain symmetry.

### Extended finite element method (X-FEM)

An ordinary finite element method (FEM) assumes that physical quantities such as stress, strain and pressure are continuous in a mesh. Thus, an ordinary FEM cannot analyze discontinuous conditions in a mesh. In contrast to an ordinary FEM, the X-FEM allows the addition of nodes to a mesh to reproduce discontinuous physical quantities. Thus, in this study, an X-FEM is considered adequate to model crack formation and reproduce the growth of cracks penetrating meshes [8–10].

### Forming limit diagram (FLD)

**Fig. 2:**
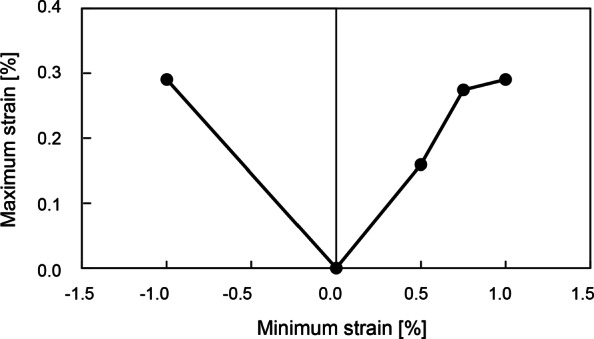
Forming limit diagram in this study

FLDs are used to predict the fracture of materials [11, 12] and show the relationship between the maximum and minimum logarithmic strains in a material when necking or fracture occurs. The forming limit on an FLD indicates the border beyond which a material breaks. When a given region shows the strain relationship above the forming limit curve, the region will break. On the other hand, the region shows the other relationship below the forming limit curve, the region will not break. There are some typical test methods to construct an FLD, but a reliable FLD for bone has not yet been reported. Hence, an FLD for glass [13] was modified for use in this study. Figure 2 and Table 3 show the FLD used in this study.

**Table 3:** Forming limit diagram data used in this study [13]

Minimum strain [*%*]	Maximum strain [*%*]
−1.00	0.290
−0.02	0.000
0.50	0.160
0.75	0.275
1.00	0.290

### Boundary conditions and loading patterns

**Fig. 3:**
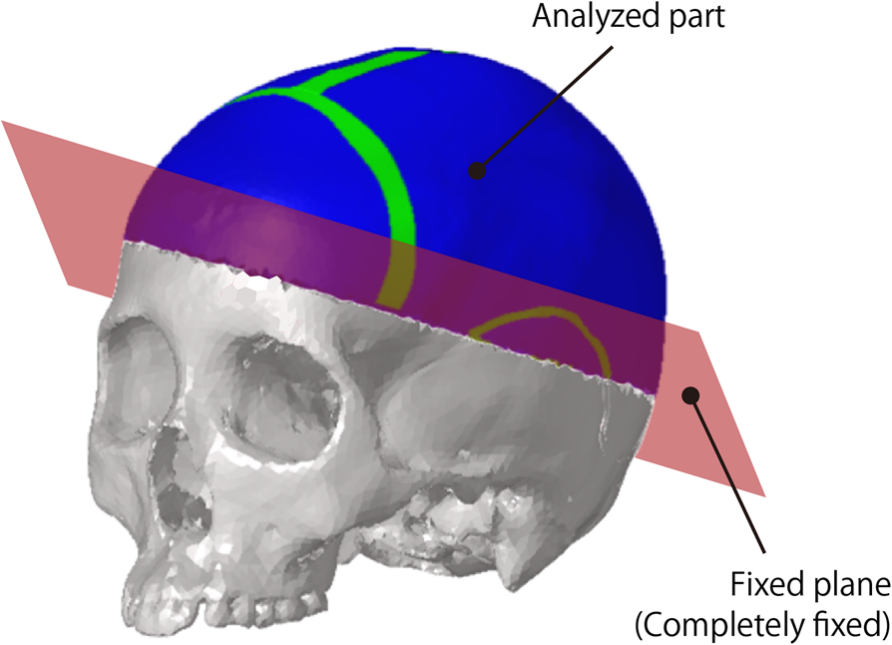
Boundary conditions in the model

In this study, to reproduce a hairline crack on the cranial bones, part of the skull was evaluated, and the edges of the part were fixed as shown in Figure 3. With this choice of computational domain, the computational time was reduced until the stress propagated to the edges. The edges were completely fixed in translations and rotations.

The skull model was struck with an impactor so that the loading conditions were similar to a previous experimental study [5]. In that study and in the present study, the impactor (weight 1.2 kg) was dropped freely with an initial velocity of 7.2 m/s. Additionally, the impactor was modeled as a sphere with a diameter of 2 mm in the present study. The impactor in the present study had the same mechanical energy as that in the previous study, because the mass and velocity are equal to those in that study.

**Fig. 4:**
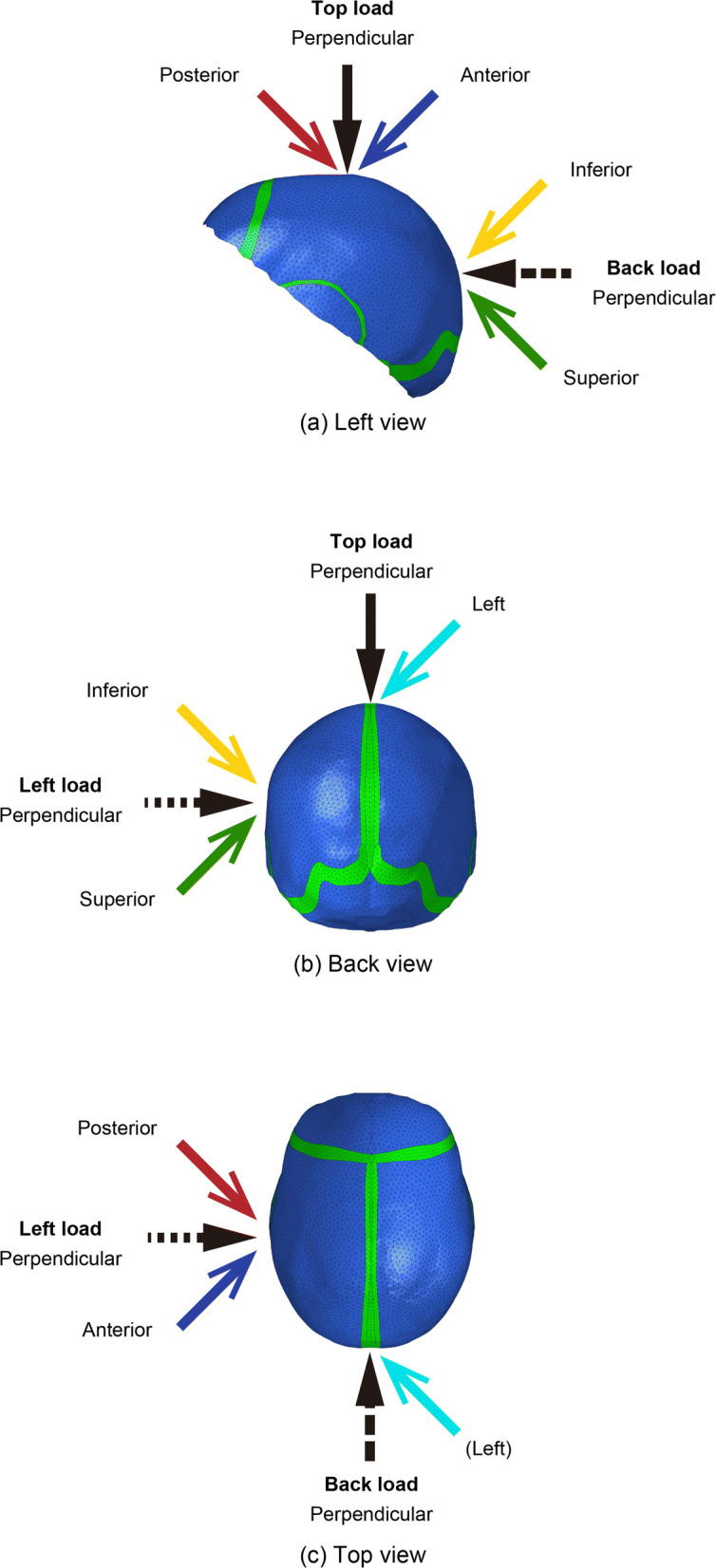
Loading conditions in the model

Figure 4 shows the loading points and loading directions in this study. The loading points are at the top, left, and back of the skull model. The loading directions included the directions perpendicular to each point and inclined by 45^∘^ with respect to the sagittal, frontal, and cronal planes. Because of the lateral symmetry of the skull model, only the leftward direction, which is inclined from the right side to the left side, was considered.

RADIOSS (Altair Engineering Inc.) was used to analyze the FE model explicitly. The time step was initially 0.1 ms, but the solver automatically changed the time step to satisfy the Courant condition.

## Results

This section presents the observed crack patterns that formed under the different loading conditions.

### Crack formation process over time

**Fig. 5:**
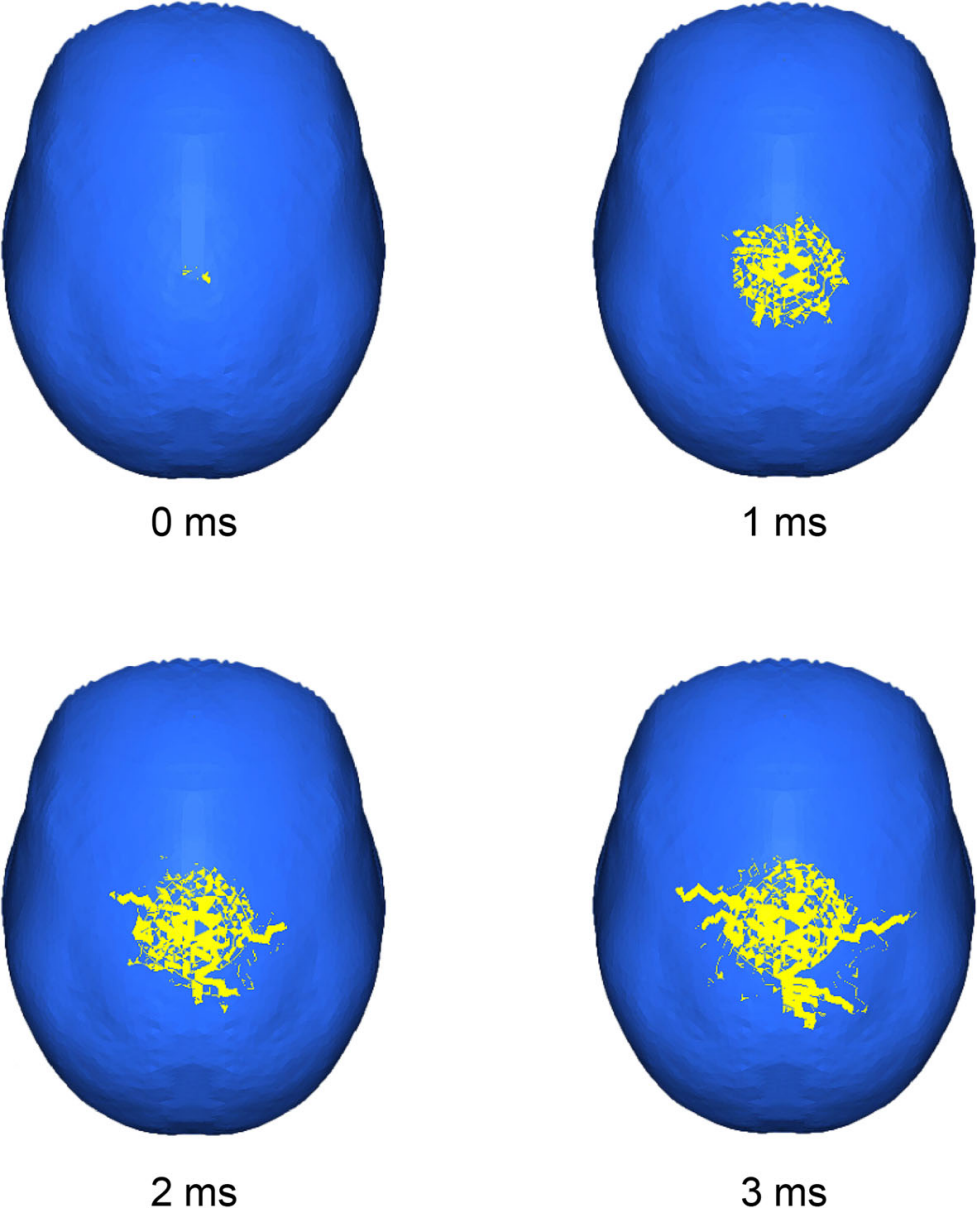
Propagation of cracks (Top, perpendicular)

Figure 5 shows the process of cracks developing from 0 to 3.0 ms at intervals of 1.0 ms with a load applied perpendicularly at the top of the skull at a time of 0 ms. When mesh elements were broken, they were removed. In the figure, the blue and yellow regions represent intact and broken(removed) elements, respectively. As shown in Fig. 5, large hairline cracks had formed by 3.0 ms. All loading conditions showed the same results over time. Namely, the durations of the crack formations finished until 3 ms regardless of the loading points or the loading direction. From these results, the relationship between the crack patterns at 3 ms and the loading conditions was evaluated.

At 1 ms, many small cracks spread in all directions. However, a few large cracks formed at 2 ms, and they developed into large cracks by 3 ms. The cracks spread bilaterally and posteriorly with almost bilateral symmetry. Generally, an explicit solution for an FE model has numerical errors, which produce asymmetric results. The present results do not necessarily demonstrate symmetry; however, they do give indications about the tendency of the shape of the cracks and the directions of the crack propagation.

### Impact at the top of the skull

**Fig. 6:**
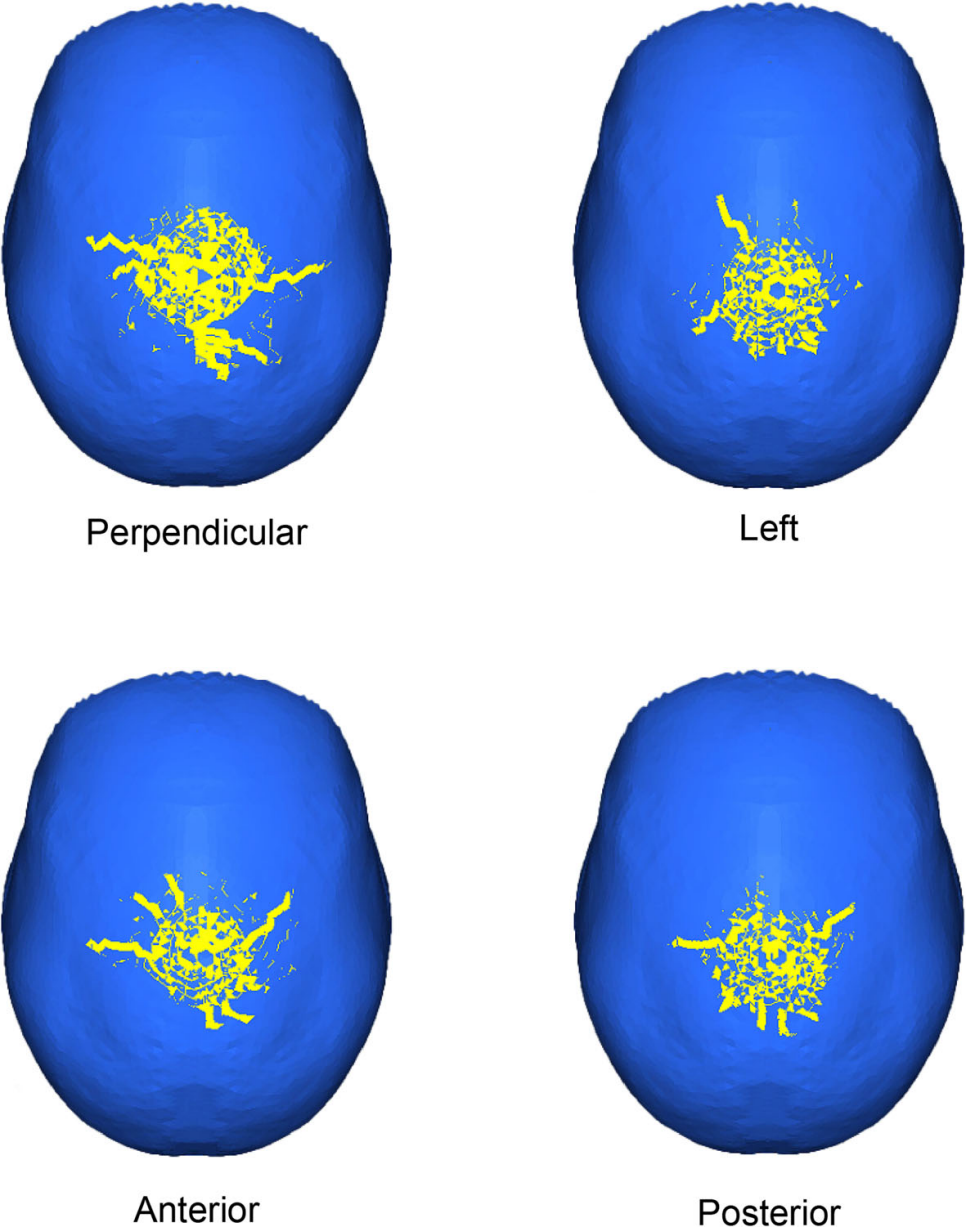
Hairline cracks under loads applied to the top of the cranium

Figure 6 shows crack patterns produced under the loads applied to the top of the cranium. As shown in Fig. 6, the perpendicular loading resulted in a hairline crack toward both the lateral and posterior directions. With the loading angled to the left, a crack also ran toward the anterior-left and posterior-left. Under anterior loading, some cracks propagated in the anterior direction, along the loading directions. However, under posterior loading, the cracks propagated toward the left and right, but not toward the posterior.

The perpendicular loading produced many small cracks that spread radially up to a distance of 30 mm, and large cracks of 30 mm developed toward both of the lateral sides and the posterior. When the load was angled to the left, the cracks developed radially in a circle with a radius of approximately 25 mm. under this loading condition, there were also large cracks propagating antero-laterally or postero-laterally with lengths of at least approximately 20 mm.

The cracks that formed under anterior and posterior loading resembled each other and showed a circular shape with a radius of approximately 25 mm. Both of these loading conditions showed large cracks that spread toward both sides but no large cracks spreading in the posterior direction. The anterior loading produced more large cracks in the anterior direction than did the posterior loading. These results demonstrate that the top loading condition could be distinguished from the resulting crack patterns.

### Impact on the left side of the skull

**Fig. 7:**
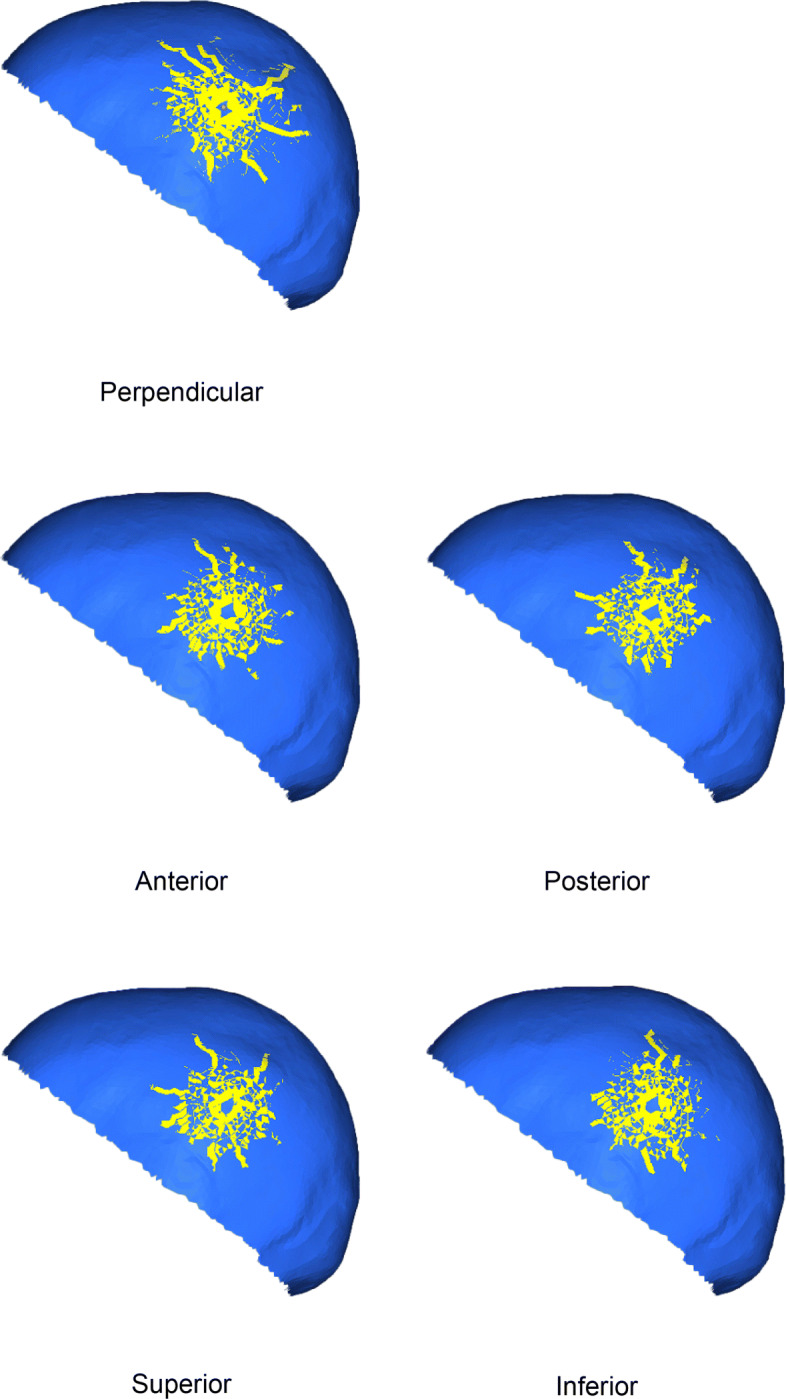
Hairline cracks under loads applied to the left side of the cranium

Figure 7 shows the crack patterns produced under the loads applied to the left side of the cranium in the five considered loading orientations. As shown in Fig. 7, the perpendicular load produced many large cracks. The anterior and inferior loadings produced similar crack patterns, with the crack lines running in the superior direction. The posterior and superior loadings also produced similar patterns, and the cracks propagated in many directions.

Under each of these five loading conditions, the hairline cracks spread radially to a distance of approximately 27 mm. Under the perpendicular loading, five large cracks of more than 20 mm in length developed in the antero-superior, postero-superior, and posterior directions. Moreover, a few thin, long cracks developed, and they were longer than the small cracks. The cracks were noticeably different from those that developed under the other four loading conditions.

The anterior and inferior loadings produced similar crack patterns. These patterns had a few large cracks (20 mm longer than the other smaller cracks) in the caudal, cephalad, anterior, and posterior directions, with the inferior loading producing additional large cracks cephalo-caudally. The posterior and superior loadings were similar to each other as well. Even though their crack shapes were also similar to the anterior case, large antero-superior and postero-superior cracks were additionally present.

These results show that antero-inferior loading and supero-posterior loading seem to produce crack patterns specific to loading direction, and these loading conditions could be distinguished based on a crack pattern. However, it would likely be difficult to distinguish between anterior and inferior loadings, or superior and posterior loadings.

### Impact at the Back of the skull

**Fig. 8:**
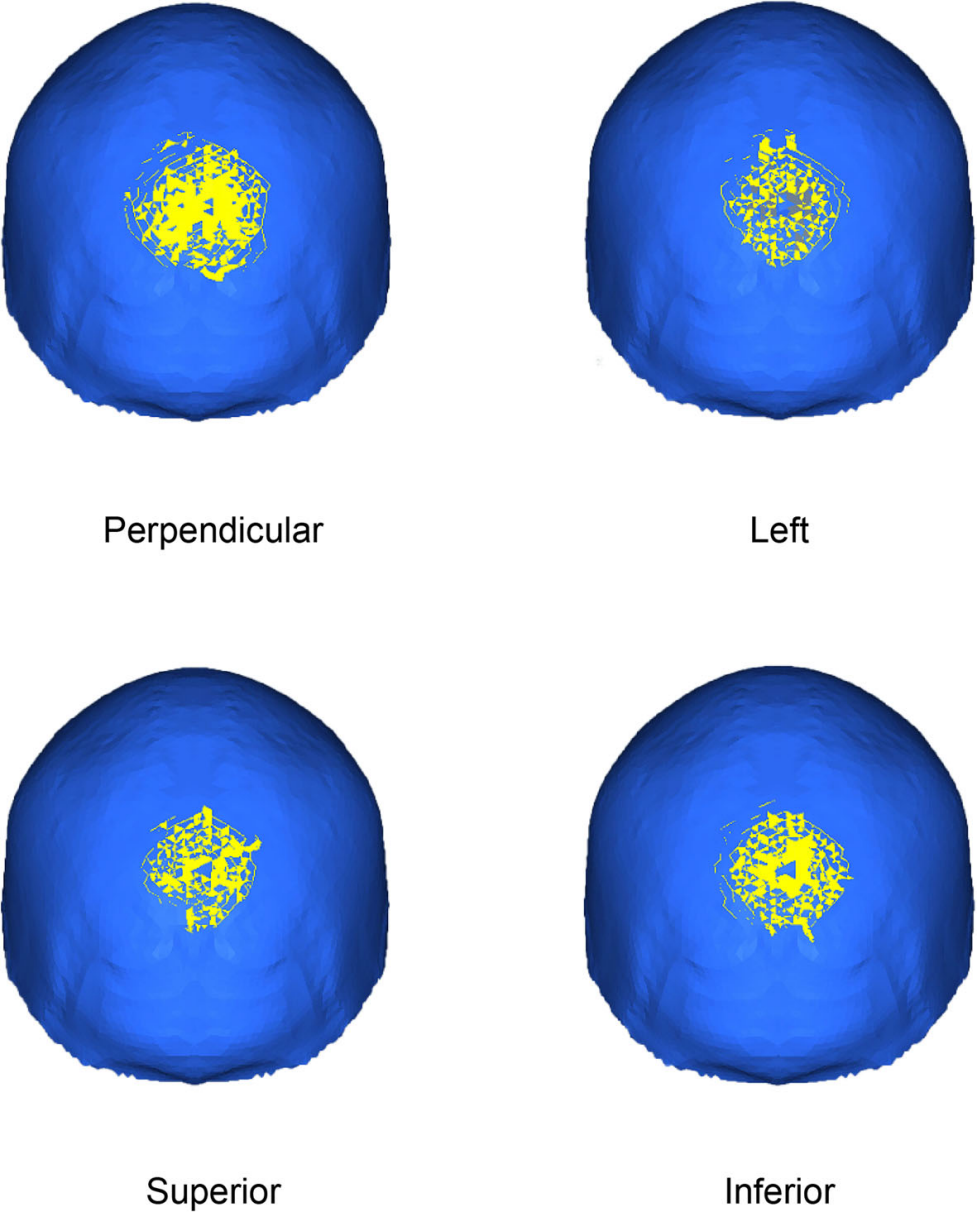
Hairline cracks under loads applied to the back of the cranium

Figure 8 shows the results with the loading applied to the back of the skull under the four considered loading conditions. None of the considered impact conditions showed any large cracks, and the small cracks propagates radially.

Under the perpendicular loading, many small cracks formed radially up to a distance of 28 mm. Although the other loading directions also showed similar cracks, they were smaller or fewer in number than the perpendicular case. The crack patterns that formed under the left and superior loadings were similar to each other and smaller than in the perpendicular case, showing no large cracks. Under the inferior loading, there were more small cracks than in the left and superior loadings.

The results of the crack patterns at the back of the skull indicate that it would likely be difficult to estimate the loading direction from the observed crack pattern. Only the perpendicular loading was clearly distinguishable from the other loading directions here, because this loading direction showed a greater number of cracks and a larger crack pattern area.

## Discussion

In this study, a total of 13 loading conditions were analyzed and various crack patterns were obtained. The results demonstrate that even identical loading locations do not necessarily generate the same crack patterns. Additionally, some loading directions can produce different crack area size or a number of large cracks, while other directions cannot. This suggests that it is necessary to simulate crack formation using a FE skull model, and that the relationship between the crack shapes and loading conditions must be further clarified in order to be able to predict the loading conditions from the crack shapes. However, this relationship might not be generalizable, and could be specific to the subject in this study. Hence, it may only be useful to apply an FE model specific to a subject.

Under all of the studied loading conditions, small cracks developed radially in a circle around a loading point. These small cracks could be affected by the FLD, in which case the FLD would control the circle size or the appearance of cracks. On the other hand, a small crack occurring from the origin resulted in numerical errors, as the small cracks seemed to be random. Therefore, small cracks should not be evaluated individually, but by clusters of the small cracks and for each loading condition. In this study, crack shapes defined by clusters of small cracks were evaluated. Even though the shapes under different loading conditions could depend on the location and direction of the loading condition, obvious and typical formations were not observed in this study. In contrast, large cracks seemed to strongly depend on the structure of the skull and loading conditions, although these large cracks were certainly also derived from small cracks.

In this study, six loading conditions resulted in symmetrical crack formations: perpendicular, anterior, and posterior loads applied to the top, and perpendicular, superior, and inferior loads applied to the left. The shapes of small cracks were almost symmetrical for all conditions, while the large cracks did not necessarily demonstrate symmetry for the loads applied to the top of the cranium. We observed dents ahead of the directions of large cracks in Figs. 6, 7 and 8. Large cracks could run toward a dent on the cranial bones. The surfaces of actual skulls generally do not have a uniform thickness and have an uneven curved surface, but the size and location of small dents vary from person to person. Even though the FE model used in this study was symmetrized, since the model was constructed from CT images, surface geometry was preserved. Therefore, the present simulation results may have resulted from the skull shape specific to the subject. However, this means that the present results might not necessarily show the general cracking patterns of the skull. Because such structural unevenness can determine the direction of large cracks, the relationship between the crack direction and the locations of dents in the skull could be investigated.

In this study, the external and internal tables and the diploe were modeled with constant thickness, while the shape, hump, and dents of the cranial bones were preserved. Before the mesh model was constructed, the geometrical data of the skull were refined and smoothed with the original geometry. Even then, the model was likely subject-specific. To eliminate individual differences and evaluate the general relationship between crack shape and loading conditions, a smoother geometry model might be useful. However, smoothing like this risks eliminating important anatomical characteristics of the skull. To avoid this problem, it will be necessary to use and compare some subject-specific FE models.

When our results were compared with the previous experimental study in the same loading condition that the impactor was struct at the top of the skull [5], the large cracks were similar to each other in the size of approximately 60 mm and the direction toward the lateral. Also, the small size cracks were within the similar radius of approximately 30 mm, even though the previous experimental study showed less number of the small cracks. Therefore, our model could have a good validity about the accuracy to estimate the crack formation.

To validate the computational method, the crack sizes in the model were compared with the stress distribution in the previous FE analysis [14]. Under the similar energy conditions of the impactor, the small cracks in this study showed the similar size to the stress concentration area in the previous analysis. Also, the directions of the large cracks had a good agreement with the stress distribution in the previous study. Therefore, our model could have an adequate validity and accuracy from the computational point of view.

In this study, the skull model was constructed based on CT images. The CT data proposed the skull shape, the width and thickness of the sutures on the skull. The anatomical information was used as the element properties. Especially, the cross-sectional structure of the skull, which consisted of the external and internal tables and the diploe, had different material properties. Therefore, our model should have the anatomical accuracy. Also, in this study, the X-FEM and FLD were embedded in the model. Using them, the model can produce the cracks at arbitrary locations and make the cracks developed toward arbitrary directions. These computational methods for FEM could reproduce the natural crack formation. Therefore, this study could improve the problems of the previous investigation.

This study had a few limitations. First, the model used here was from CT data from a single patient. The model was constructed while maintaining the anatomical structure of the skull and consisted of cross-sectional structures. However, structures specific to the subject might decrease the generality of the formation of hairline cracks of the cranial bones.

The second limitation was that the suture structure was simplified as a band. In reality, the sagittal suture follows a meandering path, and its micro-processes are complicated. However, the model did not reproduce this complex suture structure.

Finally, in this study, only the left side of the cranium was used to create the model. Neither the original subject-specific model nor a model made from the right side of the cranium was evaluated. Those models would not show the same results as in this study, and skull fractures from the different types of models would not be generalizable to each other.

Even though the present model has some limitations, the simulated results show that crack patterns may depend on the loading location and direction. By comparing our results with additional simulations, the relationship between loading conditions and the formation of hairline cracks on the cranial bones could be elucidated. Therefore, the model could be useful to estimate the loading conditions from the shapes of hairline cracks.

## Conclusions

In this study, an FE model of the skull was developed. The suture of the model consisted of the external and internal tables and the diploe. To reproduce hairline cracks, FLD was embedded and the model was analyzed by X-FEM. It was found that the size and orientation of the cracks that formed depended on the position and direction of loading. Our simulations indicate that the shapes of the cracks could be used to estimate the loading conditions. Therefore, the present simulation method and the skull model could be useful to consider the relationship between the loading conditions and hairline crack features.

## Data Availability

The datasets used and/or analyzed during the current study are available from the corresponding author upon request.

## Abbreviations

FEM: Finite Element Method
FLD: Forming Limit Diagram
